# Update on the advances and challenges in bioequivalence testing methods for complex topical generic products

**DOI:** 10.3389/fphar.2024.1330712

**Published:** 2024-02-08

**Authors:** Nedaa Alomari, Waleed Alhussaini

**Affiliations:** Department of Pharmaceutical Analysis, King Abdullah International Medical Research Center, King Saud Bin Abdulaziz University for Health Sciences, Ministry of the National Guard—Health Affairs, Riyadh, Saudi Arabia

**Keywords:** bioequivalence, testing methods, topical, generic, biowaiver

## Abstract

Most of the government regulatory agencies, including the United States Food and Drug Administration and the European Medicine Agency, demand that the generic complex topical products prove pharmaceutical and bioequivalence. The evaluation of bioequivalence for complex topical dermatological formulations is a challenging task that requires careful consideration of several factors. Although comparative clinical studies are still considered the gold standard approach for establishing bioequivalence in most formulations, these studies can be costly and insensitive to detect formulation differences. Therefore, significant efforts have been made to develop and validate alternative approaches that demonstrate bioequivalence and expedite the availability of high-quality generic topical dermatological products. This article reviews the current methods for determining the bioequivalence of topical formulations in humans, with particular emphasis on recent advances in these methodologies. Most of the alternative methods are sensitive and reproducible, with the capability to ease the financial burden of comparative clinical studies within a short delivery time. The limitations associated with each technique are reviewed in detail.

## 1 Introduction

Topical drug formulations are primarily designed to deliver drugs to certain skin layers. These formulations include creams, gels, and ointments. The pharmaceutical market has many topical innovator brands without generic products due to the complexity associated with generic drug development and assessments (Li et al., 2018). In assessing topical generic formulations, the regulatory authorities request a demonstration of the pharmaceutical and therapeutic equivalence to establish bioequivalence (BE) between the new generic product and the reference-listed drug (RLD) (Shah et al., 2015). BE studies may be conducted based on the performance of comparative clinical trials, pharmacokinetic (PK) measurements, pharmacodynamic (PD) measurements, and *in vitro* studies (Jalali and Rasaily, 2018). Although comparative clinical trials are the gold standard for establishing BE, they usually require many subjects (n > 500) and must have static relevance due to complex factors that could impact skin permeation. Consequently, these studies are known to be the most expensive part of the research and development of topical generic products. Regulatory agencies may approve scientifically valid alternative methods to establish BE “for a drug that is not intended to be absorbed into the bloodstream,” which can detect significant differences between the generic product and the RLD (Food and Administration).

Many scholars have stated that comparative clinical trials are less sensitive as a method for demonstrating BE (Yacobi et al., 2014; N Dri-Stempfer et al., 2009; Navidi et al., 2008). These limitations have encouraged regulators to explore alternative *in vitro* and *in vivo* approaches for BE assessment of generic topical drugs and improve the current approaches. The objective of this review article is to summarize most of the BE testing methods for complex topical generic products. The review discusses the strengths and limitations of the most promising approaches and focuses on the suitability of these methods in the demonstration of BE. It is expected that advanced techniques in the field of pharmaceutical sciences will facilitate the development of robust BE methods for these complex dosage forms and enable more efficient, accurate, and simple approaches to accelerate the availability of high-quality generic topical products.

## 2 Topical drug classification system

The proposed topical drug classification system (TCS) distinguishes generic formulations from the RLD based on the qualitative (Q1) and quantitative (Q2) equivalence of the composition of the complex topical dosage forms and the similarity of *in vitro* release (IVR), which reflects microstructural sameness (Q3) (Shah et al., 2015). Four different classes are presented for various scenarios that may arise, considering whether a biowaiver is appropriate. In the case of TCS class 1, all three parameters, Q1, Q2, and Q3, are the same between the RLD and generic product. In this case, the generic product may be suitable for a biowaiver. If they are not the same, then a biowaiver cannot be provided, and additional studies will be required (Shah et al., 2016). In TCS class 2, generic products have the same Q1 and Q2 as the RLD but different Q3. In this case, the generic product is not eligible for a biowaiver, and the applicant must ensure that the safety and efficacy profiles of the product will not be impacted as per agency requirements. In TCS class 3, there are Q1 or Q2 differences between the test product and the RLD, and additional *in vitro* studies may be required, such as the excipient component evaluation (Shah et al., 2015). However, the product has a similar release from the vehicle and delivery through the skin, resulting in Q3 similar to that of the RLD. These generic products will be eligible for a biowaiver. In the case of TCS class 4, the generic product is different in composition, resulting in a different microstructure (Buhse et al., 2005).

**FIGURE 1:**
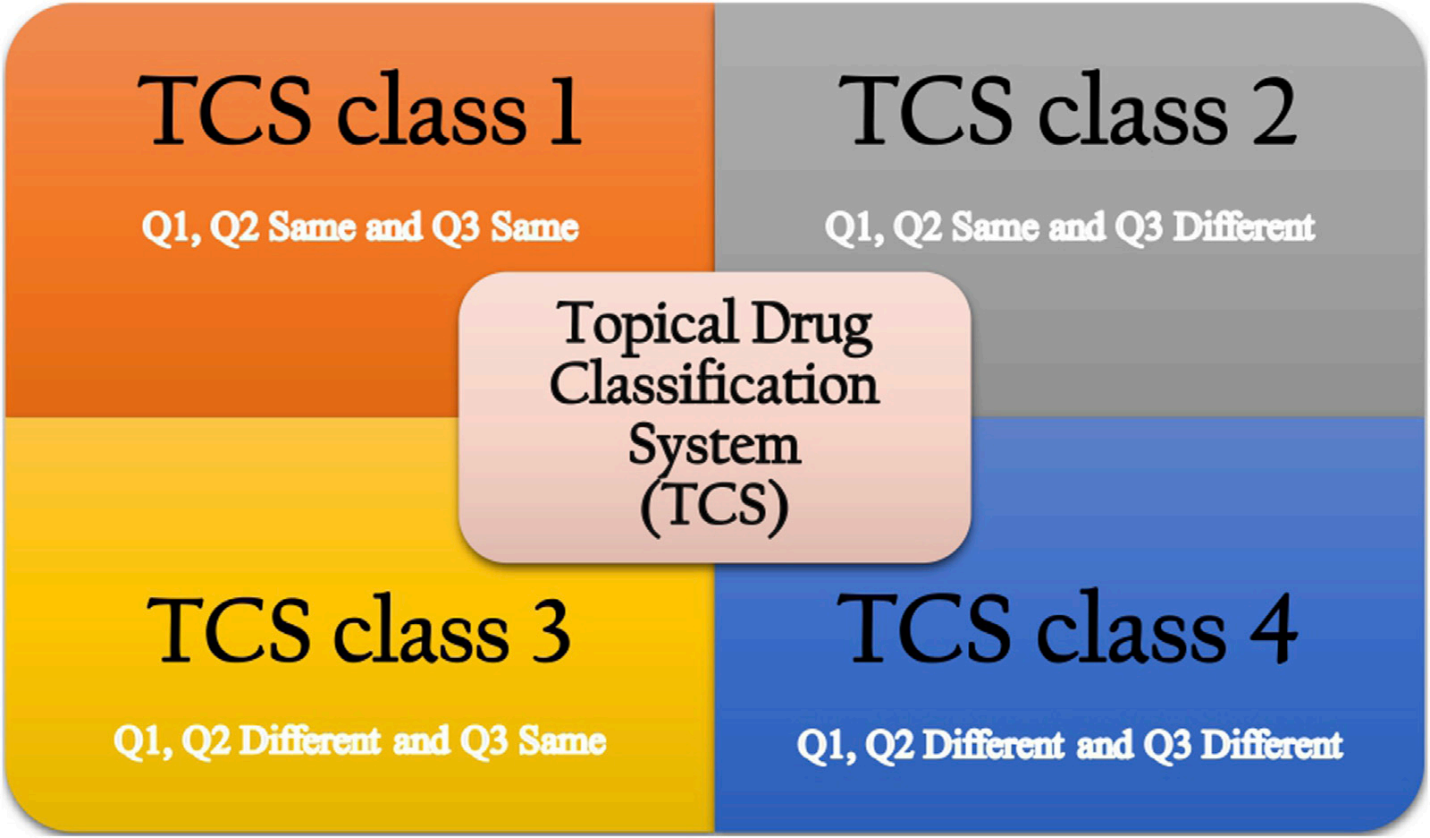
Topical drug classification system (Shah et al., 2015).

In brief, according to the TCS classification, generic topical drug products in classes 1 and 3, which have similar profiles to the RLD, will be eligible for biowaivers. Generic products in classes 2 and 4 must conduct additional *in vivo* studies to demonstrate BE. A schematic diagram of the TCS is presented in Figure 1. Additionally, the regulatory requirements for documenting the BE of topical products differ between the United States Food and Drug Administration (US FDA) and the European Medicines Agency (EMA). The US FDA drafts specific guidance for each product. In contrast, the EMA prefers a “one-size-fits-all” approach. Several recent publications have discussed the suitability of each draft guideline criterion in detail and compared the agencies’ requirements (Ilić et al., 2021; Miranda et al., 2022; García-Arieta et al., 2023).

## 3 Methods

### 3.1 Comparative clinical endpoint study

Comparative clinical endpoint studies are the traditional approach to demonstrate BE to the RLD for most complex drug products, such as creams, gels, and ointments. These studies are often designed as randomized, crossover, or parallel studies using a prospective generic drug product (U.S. FDA a). Although clinical endpoints provide direct therapeutic outcomes, they are also associated with several challenges. Clinical endpoints can be confounded by several factors, including high variability and insensitivity due to environmental and pathophysiological factors. In addition, the weak therapeutic efficacy of some topical drugs and unstandardized doses can make testing challenging (Carroll et al., 2004; Krejci-Manwaring et al., 2007). These factors can influence the performance of a given topical product and make such studies less efficient and less reliable. Generally, comparative clinical trials are tedious to perform, costly, require large patient populations, and may not efficiently detect formulation variabilities. For these reasons, clinical scientists recommended developing standard methods that are more reliable and sensitive surrogates for the BE assessment of topical formulations (Chen et al., 2011; Harris, 2015).

### 3.2 Pharmacodynamic study (vasoconstriction assay)

An *in vivo* pharmacodynamic study, also called a vasoconstriction assay (VCA), is limited to topical corticosteroid (also known as glucocorticoid) products (Chang et al., 2013a). When applied topically, corticosteroids produce a visible skin-blanching response caused by vasoconstriction (Wiedersberg et al., 2008). The blanching methodology, commonly referred to as the Stoughton–McKenzie assay, employs the visual assessment of blanching to evaluate the BE of topical formulations in healthy volunteers based on a 0–4 intensity scale evaluated by professional observers or via a quantitative method, such as thermography, reflectance spectroscopy, or laser Doppler velocimetry (Aiache et al., 1980; Ryatt et al., 1982; Andersen et al., 1993). The choice of the assessment model of visual blanching or chroma meter is discussed in the guidance (Singh et al., 1999). The pharmacodynamic effect has been correlated with the clinical efficacy of topical corticosteroid products. Thus, the FDA currently accepts a VCA as a surrogate method to evaluate the BE of glucocorticoid topical dosage forms (Chang et al., 2013a; Chang et al., 2013b).

Although the method requires fine-tuning, the pharmacodynamic approach works well with semisolid formulations. A VCA is inexpensive and requires fewer healthy volunteers than clinical endpoint studies. Although a VCA is accepted by various regulatory authorities, from a practical perspective, several limitations impact the intensity of skin blanching, mainly the drug vehicle, concentration, location, posture, and occlusion. The influence of vehicles based on their characteristics, for example, high solubility and rapid delivery, enhances drug response. Therefore, the vehicle may impact the intensity of skin blanching. The uptake level of a topical formulation depends on the concentration and duration of application, which may influence the intensity and location of skin blanching. The poorest blanching usually occurs within 1–4 cm of the wrist and elbow. In addition, occlusive ingredients tend to show better drug penetration and greater blanching intensity (Schwarb et al., 1999; Fesq et al., 2003; Görne et al., 2007). Despite the high variability of the assessment, this method has been successfully standardized and recommended for use by the FDA when applying for a biowaiver for glucocorticoid-containing topical products.

### 3.3 Pharmacokinetic study

Pharmacokinetic analysis can be performed in special cases to evaluate BE for topical formulations where the drug displays significant plasma/tissue levels, similar to the BE assessment of oral dosage forms. A rare example of pharmacokinetic application includes the draft guidance issued by the FDA for lidocaine patches, followed by an approved drug (ANDA; Nalamachu and Gudin, 2020). The pharmacokinetic study is mostly used to establish the safety profile of topical products, where the level in plasma is significant and in line with the concentration at the site of action. However, the application of this technique in BE assessment is very restricted.

### 3.4 Tape stripping (dermatopharmacokinetics)

Tape stripping (TS), also termed dermatopharmacokinetics (DPK), is a noninvasive approach to assess the local bioavailability or BE in the *stratum corneum* (SC) layer using an adhesive tape. This technique can be applied *in vivo* and *in vitro* using skin models, including humans or animals such as pigs, rats, and mice. After topical application and penetration of formulations, the basic process of TS is involves placing adhesive tape on multiple sites on the skin, followed by applying pressure using a roller or pressure device. The adhesive tape is then removed and placed in a vial to avoid folding. Then, the product concentration on each tape is quantified individually. Penetration profiles may be reported as an indicator of depth within the skin layer. The data obtained could be used for evaluating different topical formulations to establish BE (Escobar-Chavez et al., 2008). The rationale behind the TS approach is that products targeting the SC layer must penetrate and reach deeper skin layers, and the consecutive removal of SC layers allows this technique to evaluate topical products in different layers of the skin (Russell and Guy, 2009). The DPK method was under review by the FDA, and a draft guideline was issued to establish BE for topical formulations in 1998. However, due to contradictory results obtained from independent laboratories regarding two generic tretinoin products, the guidance was withdrawn in early 2002 (Mohan and Wairkar, 2021).

TS is a simple, robust, and minimally invasive method. Given its simplicity, TS can be used in various research areas, such as multiplex immunoassays and biomarker identification. However, several limitations could affect the quality of the assessment. First, the DPK method is only valid for drugs whose site of action is the SC. Second, the current guidance lacks clear instructions on the amount or depth of the SC that should be collected and analyzed. Third, and perhaps the most complex limitation, the guidance requires a complex validation process involving many participants and replicate application sites. In addition, there are technical factors that could eventually alter the outcomes of the assessment. These factors, which include skin surface contamination, must be addressed by limiting the use of topical products prior to the experiment. Unfortunately, inconsistencies in the TS procedure may lead to an uneven amount of product being absorbed into the SC.

### 3.5 Dermal microdialysis

Dermal microdialysis (DMD) is an *in vivo* sampling technique that can analyze the cutaneous pharmacokinetics and concentrations of pharmaceutical products in dermal tissue. DMD sampling is also called dermal, intradermal, or cutaneous microdialysis. When a topical formulation is applied to the skin, the unbound products in the dermal interstitial fluid (ISF) diffuse into the lumen of the membrane due to a concentration gradient. The concentration of the drug in the extracellular space of the skin can be measured using a thin probe containing small perfused membrane systems filled with a skin-compatible sterile buffer, which collects samples within the dermis layer (Anderson et al., 1991; Benson and Watkinson, 2012). A critical advantage of this technique is its capacity to measure topical products penetrating both healthy and diseased skin (Gao et al., 2014). The method is quite inexpensive because the probes can be easily manufactured. However, DMD has a certain intrinsic limitation: its relatively invasive technique may induce inflammation (Kreilgaard et al., 2001). It is also necessary to use associated, suitable internal standards for drug concentration determination (Ao and Stenken, 2006). A recent study by Senemar et al. (2019) corroborated the feasibility of using DMD to establish the BE of topical products containing metronidazole by comparing the area under the curve (AUC). DMD was sufficient to discriminate differences in bioavailability between different formulations with a power greater than 90%, providing scientific evidence that DMD can be used as a promising alternative for comparative clinical endpoint trials.

### 3.6 Dermal open-flow microperfusion

Dermal open-flow microperfusion (dOFM) is a sampling technique that allows the evaluation of dermal PK and PD parameters via continuous sampling of ISF from the dermis. The main advantage of dOFM over the DMD is the 0.55-mm-diameter probe design, an open mesh made from polyetheretherketone (PEEK) that enables direct sampling of high molecular weight compounds and provides access to the tissue milieu directly (Schaupp et al., 1999). The main strength of dOFM is its ability to assess any topical drug regardless of its molecular size, lipophilicity, or the formulation that is used for its delivery (Bodenlenz et al., 2016; Kolbinger et al., 2017; Birngruber et al., 2022). dOFM studies have demonstrated low intra-subject variability, and none of the methodological factors contributed to that variability (Bodenlenz et al., 2020). Furthermore, due to the highly standardized dOFM setup, BE can be determined with a smaller number of healthy participants (20–30) than in comparative clinical endpoint studies (hundreds to thousands of participants) for long sampling intervals using the wearable pumps (Zhu and Sun, 2019; Birngruber et al., 2022).

### 3.7 *In vitro* release testing

*In vitro* release testing (IVRT) is a valuable tool that can compare the *in vitro* release rates of the test and RLD. IVRT analyzes the drug after its release from the vehicle into the receptor medium, which is separated by a synthetic membrane (Rath and Kanfer, 2020). Many diffusion systems have been used for IVRT, such as flow scatter, immersion cell, Franz, vertical, horizontal, static, and side-to-side diffusion cell systems (KHATANA et al., 2022). A Franz cell or vertical diffusion cell (VDC) system employing cadaver skin is well known in the pharmaceutical industry and among drug researchers working in the dermatology field. IVRT is a well-established and sensitive method that can reflect changes in the physicochemical properties of topical products, such as drug solubility, rheological properties, and particle size. IVRT is an easier BE assessment approach than *in vivo* testing. Continuous sample collection is not necessarily required as its operation can be automated, and only a small amount of product is needed (Supe and Takudage, 2021). IVRT provides a benefit compared to the manual diffusion method because it provides fast, reproducible, and accurate sampling. A recent study by Tiffner et al. (2018) highlighted the essential components of the test system, with a focus on IVRT parameters and specific acceptance criteria.

### 3.8 *In vitro* permeation test

An *in vitro* permeation test (IVPT) is also called an *in vitro* skin penetration test, and it provides a cost-effective BE assessment of topical drugs. Typically, *ex vivo* human skin is used in combination with cell diffusion techniques that are considered the optimal techniques for assessing skin pharmacokinetics (Yacobi et al., 2014). IVPT is recognized by the FDA and the EMA as a regulatory method for determining the BE of locally acting topical drugs. Numerous studies have demonstrated that correctly implemented IVPT methodologies can yield the same results as *in vivo* clinical endpoint studies for the BE of two semisolid drugs. Lehman and Franz (2014) concluded that IVPT with cryopreserved human skin was more accurate and less variable than an equivalent pharmacodynamic study and strongly supported the application of IVPT in determining the BE of complex topical products. Moreover, a novel experimental design for the generic acyclovir 5% cream was published based on FDA guidance to reduce the sample number required to establish BE (Lim et al., 2023). The recently accepted strategy for the assessment of BE in topical products using the IVPT technique is only applicable to drugs that rapidly penetrate human skin, allowing for the evaluation of drug permeation profiles.

### 3.9 Confocal Raman spectroscopy

Confocal Raman spectroscopy (CRS) is a noninvasive approach that has been widely used in human skin research. CRS allows for real-time monitoring of topical product penetration through the skin (Caspers et al., 1998; Chrit et al., 2007). CRS quantifies the rate and extent of substance uptake and its clearance via a laser beam directed at the skin layer (Kezic, 2008). Distinct Raman spectra are recorded for specific molecules. CRS offers a nondestructive, accurate, and reproducible method for obtaining a topical product’s bioavailability. However, a major challenge of CRS is the lack of absolute quantification capabilities (Herkenne et al., 2008). Recently, several proof-of-concept studies applied CRS to measure topical product concentration. Iliopoulos et al. (2023) explored the feasibility of the CRS method to assess the BE of topical products. The permeation of two marketed ibuprofen gel formulations was investigated *in vivo*. Mohammed et al. (2014) studied the *in vitro* permeation of seven different niacinamide vehicles with *in vivo* uptake in human skin models. The results demonstrated that the niacinamide signal was directly proportional to niacinamide permeating results *in vitro.* These studies suggested that CRS can validate the BE of dermal drugs.

### 3.10 Near-infrared spectroscopy

Near-infrared spectroscopy (NIRS) has been widely used as a noninvasive approach to assess product permeation across the skin layers. The principle behind NIRS is as follows: near-infrared (NIR) waves can penetrate the skin and quantify product diffusion by measuring the corresponding IR absorption, followed by linear multivariate statistical computation (Mak et al., 1990). NIRS is an advanced, rapid, and noninvasive technique that provides *in vivo* real-time monitoring signals. NIRS is superior to other approaches due to its capability to analyze volatile drugs. For these reasons, the FDA has provided guidance documents for the development, validation, and use of NIRS in 2021. Medendorp et al. (2006) monitored the *in vivo* concentration of several products in hairless guinea pig skin using NIRS. SAKIRA et al. (2021) used NIRS combined with multivariate data analysis to develop chemometric models for the classification and quantification of metronidazole. Taken together, NIRS and CRS are promising techniques due to their nondestructive properties. Their future applications in complex topical BE assessments need to be optimized.

### 3.11 In vitro–in vivo correlation

The FDA defines in vitro–in vivo correlation (IVIVC) as “a predictive mathematical model describing the relationship between an *in vitro* property of a dosage form and a relevant *in vivo* response” (Lu et al., 2011). The establishment of IVIVC has been promising in the fields of pharmaceutical product development and BE, aiming to use *in vitro* drug release performance to predict *in vivo* drug behavior. Various drug parameters such as physiological, physicochemical, and biopharmaceutical properties must be taken into consideration to establish an IVIVC of topically applied drug products, as well as *in vitro* parameters, such as IVRT or IVPT and *in vivo* parameters, such as data obtained from TS or VCA (Shah, 2005). Until now, no regulatory IVIVC guidance for a topical complex formulation has been drafted. However, the principles described in the FDA’s documents for ER oral dosage forms have been used to establish IVIVC for topical products. Generally, establishing an IVIVC model involves mathematical modeling and data analysis, which are reviewed in detail by Lu et al. (2011). A successfully developed IVIVC model can be used as a surrogate method for BE studies to obtain a biowaiver. IVIVC can also be used as a guide during topical formulation product development to ensure the safety and efficacy of the final product. Although establishing a meaningful IVIVC for complex topical products is quite challenging due to the complexity of BE processes, one recent study has shown promising results. A study conducted by Rath and Kanfer (2023) established the IVIVC of topical metronidazole creams following IVRT and TS in healthy human participants, respectively, with good qualitative and quantitative correlations for the reference and test products.

### 3.12 Physiologically based pharmacokinetic modeling

Physiologically based pharmacokinetic (PBPK) modeling and simulation are quantitative approaches that can predict the pharmacokinetic profile of the active ingredient in humans. The method considers many intrinsic (e.g., age, genetics, and organ dysfunction) and extrinsic (e.g., drug–drug interactions) factors in a mechanistic manner. More specifically, dermal PBPK models describe skin permeation at or near the site of action and support alternative BE approaches through virtual screening of healthy individuals and patients in special populations (e.g., pediatric and pregnant populations) at the regulatory level (Hamadeh et al., 2021; Yun et al., 2022). The particular advantage of the PBPK models in the context of topical formulations lies in their ability to include inter- and intra-subject variability in skin physiology parameters such as skin thickness, blood flow, and skin pH.

PBPK models utilize a computer simulation system that reduces time and associated costs compared with a comparable BE study and have been extensively used during generic development, construction of a safe space, and approval processes. Virtual BE studies have been carried out to speed the generic filing for a biowaiver on a case-by-case basis (Tsakalozou et al., 2021). The FDA has recognized the role of PBPK modeling and simulation as an alternative BE method, and the first grant in the field of topical dermatological drugs was awarded in 2014, with several to follow (Lionberger, 2019; Zhao et al., 2019). On 16 May 2019, the FDA granted the first abbreviated new drug application (ANDA) approval for generic diclofenac sodium topical gel 1%, where the applicant incorporated a PBPK model to demonstrate BE (Tsakalozou et al., 2021). A collaborative scientific effort is required to further enhance these PBPK modeling and simulation methodologies for complex topical dermatological products.

## 4 Future opportunities

In this scientific review, we have explained the progress of several promising alternative BE approaches for topical dermatological products and their limitations, which are summarized in Table 1. The high cost of comparative clinical endpoint studies makes it necessary to develop efficient approaches that may facilitate the development, registration, and approval of complex topical products. The future direction of BE assessment for topical products would be toward the refinement and standardization of the existing methodologies for regulatory purposes. Additionally, the integrated use of multiple approaches may be sufficient to demonstrate BE, as the limitations of these approaches are not identical. In conjunction with *in vitro* studies, the use of PBPK modeling may adequately benefit studies with detailed characterization for topical dermatological products.

**TABLE 1:** Summary of advances and limitations of bioequivalence testing methods for complex topical generic products.

Method	Advantage	Disadvantage
**Comparative clinical endpoint study**	• Consonantly required for topical BE assessment	• High variability
	• Eliminate the need for further studies (Yacobi et al., 2014)	• Costly and time consuming (Yacobi et al., 2014)
**Pharmacokinetic trials**	• Provides safety profile (Chow, 2014)	• Limited application (Chow, 2014)
**Vasoconstriction assay**	• Cost effective (Lehman and Franz, 2014)	• High variability
		• Limited to corticosteroid drugs (Lehman and Franz, 2014)
**Dermal microdialysis**	• Real-time monitoring (Holmgaard et al., 2010)	• Limited to short-duration studies
		• Required experience personnel
		• Minimal invasiveness (Holmgaard et al., 2010)
**Tape stripping**	• Efficient and simple method	• Lack a standardized method
	• Painless and allows multiple sampling (Au et al., 2010)	• Not ideal for volatile chemicals (Au et al., 2010)
**Dermal open-flow microperfusion**	• Reduction of inter-subject variability	• Short sampling time (Bodenlenz et al., 2017)
	• Real-time continuous data monitoring	
	• Can be used with patients experiencing dermatologic diseases (Bodenlenz et al., 2017)	
***In vitro*** **release testing**	• High sensitivity	• Limited correlation to *in vivo* studies (Dandamudi, 2017)
	• Simple and easy to use method (Dandamudi, 2017)	
***In vitro*** **permeation test**	• Suitable for micro sampling	• Biological variability
	• Highly sensitive (Lehman and Franz, 2014)	• Assay complexity (Lehman and Franz, 2014)
**Confocal Raman spectroscopy**	• Noninvasive	• Low penetration depth
	• Real-time penetration monitoring (Iliopoulos et al., 2023)	• Long acquisition times (Iliopoulos et al., 2023)
**Near-infrared spectroscopy**	• Noninvasive	• Complicated model development (Narkar, 2010)
	• Real-time penetration monitoring (Narkar, 2010)	
**In vitro–in vivo correlation**	• Cost reduction	• Model complexity (Burmeister Getz et al., 2021)
	• Improvement of product quality (Burmeister Getz et al., 2021	
**Physiologically based pharmacokinetics**	• Decreases the reliance on human trials	• Model complexity (Tsakalozou et al., 2021)
	• Cost-effective method	
	• Predicts drug–drug interactions. (Tsakalozou et al., 2021)	
